# Unique indolizidine alkaloid securinine is a promising scaffold for the development of neuroprotective and antitumor drugs

**DOI:** 10.1039/d1ra02558a

**Published:** 2021-05-26

**Authors:** Sergey Klochkov, Margarita Neganova

**Affiliations:** Institute of Physiologically Active Compounds Russian Academy of Sciences Chernogolovka Russia klochkov@ipac.ac.ru +7(496)-524-2650 +7(496)-524-2650

## Abstract

Alkaloids, secondary plant metabolites, are used in traditional medicine in many countries to treat various pathological conditions. Securinine, a unique indolizidine alkaloid combining four cycles, “6-azobicyclo[3.2.1]octane” as a key structure fused with α,β-unsaturated-γ-lactone and piperidine ring, has a broad spectrum of actions including anti-inflammatory, antibacterial, neuroprotective and antitumor, and has been previously used in medical practice. It has several reactive centers, which are double bonds at positions 12–13 and 14–15, and this is a challenging scaffold for the synthesis of biologically active compounds. In this review, works on the production of modified securinine derivatives and their biological activity are addressed. Both monovalent and bivalent derivatives that are most promising in our opinion, and have potential for further research, are considered.

## Abstract 2

Derivatives of unique indolizidine alkaloid securinine used for neuroprotection and as antitumor agents.

## Graphical abstract

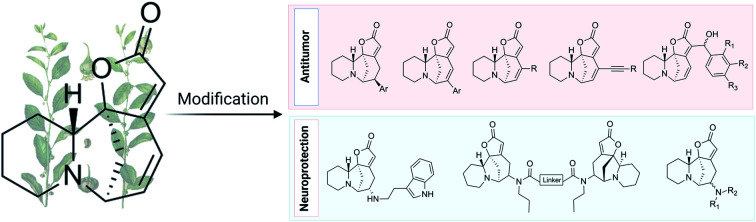

## Introduction

**Fig. 1:**
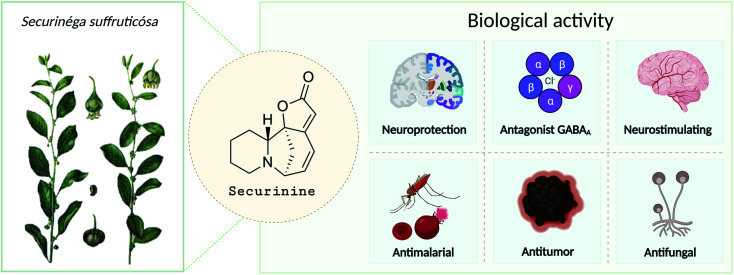
Target types of securinine's biological activity in therapy of different disorders.

Securinine (1) was isolated in 1956 from *Securinega suffruticosa* (Pall.) Rehder.^1^ The studies have shown that it is the main alkaloid present in the roots of plants belonging to the genus *Phyllanthus*, *Securinega*, and *Flueggea*.^2–6^ Securinine has a pronounced biological activity (Fig. 1) and has been clinically used in some countries.^6,7^ Further study has shown that securinine exhibits a wide range of biological properties including acetylcholinesterase inhibiting activity,^8^ antimalarial and antimicrobial activities,^9,10^ and antifungal activity.^11^ Securinine is also characterized as a powerful stimulant of the central nervous system; at a dose level of 0.1–0.2 mg kg^−1^, it has a strong spastic effect, similar to the actions of strychnine when used at a dose of 5–30 mg kg^−1^.^1^ Moreover, securinine in a number of experiments improved the learning of experimental animals.^12^

The initial securinine (1) research focused on its CNS (central nervous system) activity. It is a selective antagonist of GABA (gamma aminobutyric acid) receptors.^13,14^ It has been shown that securinine can be used in the treatment of neurological diseases such as amyotrophic lateral^15^ and multiple sclerosis.^13,16^ In ref. 17, securinine is considered as a promising anti-inflammatory and neuroprotective drug in the treatment of Parkinson's disease. It has been shown that securinine (1) inhibits the activation of the inflammatory mediator of the transcription factor NF-κB, as well as its activator ERK. In addition, it inhibits iNOS expression and NO production, both of which are also activated by NF-κB. Securinine (1) also showed a neuroprotective effect on primary dopaminergic neurons in an *in vitro* model of Parkinson's disease *via* the inhibition of lipopolysaccharide-induced microglial activation. Lin *et al.* describe securinine (1) as a potential neuroprotective agent for the treatment of Alzheimer's disease (AD). Chronic administration of securinine prevented the manifestation of cognitive impairments in rats caused by the administration of the amyloid fragment (Aβ_25–35_).^8,18,19^ It may significantly reduce the inflammatory response in glial cells caused by the action of β-amyloid protein.

In the last two decades, the antitumor activity of securinine has also been extensively studied. It has been found that securinine stimulates apoptosis in p53 knockout colon cancer cells,^20^ as well as in human breast cancer MCF-7 cells,^21^ human promyelocytic leukemia cells HL-60,^22^ human promyelocytic leukemia cells K-562,^23^ HCT 116,^20^ SW480 (ref. 24 and 25) colon cancer cells, HeLa cervical cancer cells,^26^*etc.*

The study of the antitumor action of securinine revealed the multitarget nature of its action. It has been shown that mitochondrial dysfunction, ROS generation, as well as mitogen-activated protein kinase (MAPK) activation are some of the molecular mechanisms of apoptosis activation under the action of securinine.^21,27^ A number of studies have shown the modulating effect of securinine on the PI3K/AKT/mTOR signaling pathways, a decrease in the expression level of Bcl-2, mTOR, and P70S6k and the effect on the overexpression of several proapoptotic proteins, such as Bax.^21,22,25^ Cell treatment with securinine resulted in the arrest of the cell cycle in the G2/M phase in *p53* knockout HCT 116 cells.^20^ Several studies have shown the ability of securinine to induce apoptosis *via* the activation of 3 and 7 caspases.^24^ It has also been shown that incubation of acute myeloid leukemia (AML) cells with securinine leads to their differentiation by activating DNA damage signaling, which was confirmed in *in vivo* experiments on nude mice.^28^

**Fig. 2:**
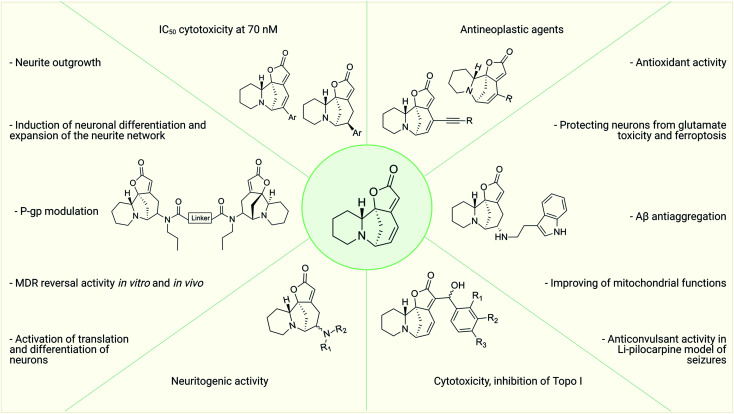
Variants of chemical modification of the natural alkaloid securinine to increase the biological activity.

**Fig. 3:**
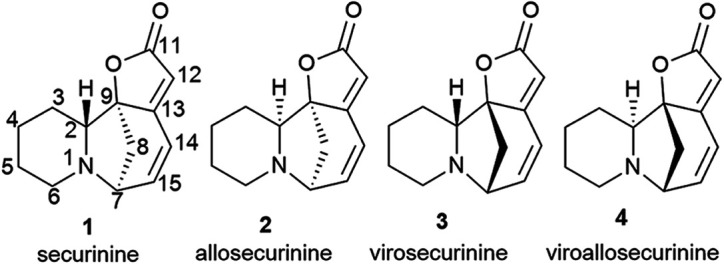
Natural securinine alkaloids.

Thus, over the past decades, the perspective of securinine used as a basis for the development of both neuroprotective and anticancer drugs has been conclusively shown (Fig. 2).^22,24,29–31^ Securinine (1) is a unique indolizidine alkaloid combining four rings, “6-azabicyclo[3.2.1]octane” as a key structure fused with α,β-unsaturated-γ-lactone and piperidine ring, and is a rigid molecule.^13^ In nature, there are four securinine isomers (Fig. 3), differing in the ring configuration at position 7-α, 9-α (securinine 1 and allosecurinine 2, levorotatory) and 7-β, 9-β (virosecurinine 3 and viroallosecurinine 4, dextrorotatory).^32^

As the structure of the natural alkaloid securinine 1 shows, it has several reactive centers: first of all, these are double bonds at positions 12–13 and 14–15. Therefore, the use of the securinine scaffold for the synthesis of derivatives, and potential neuroprotective and anticancer drugs focuses on these reaction centers.

## Literature search strategy

A systemic search of electronic databases including the PubMed, Scopus, Web of Science, Science Direct, and Google Scholar for papers reporting on securinine and their derivatives as well as bioactivity of compounds was conducted. The data were analyzed from 1956, when securinine was discovered, to the present. The review included only published articles and did not consider unpublished works and non-peer reviewed articles. Language restrictions were implemented and only articles in English were included.

## Reactions at the C14–C15 double bond

### Synthesis of allomargaritarine and amino derivatives

**Scheme 1:**
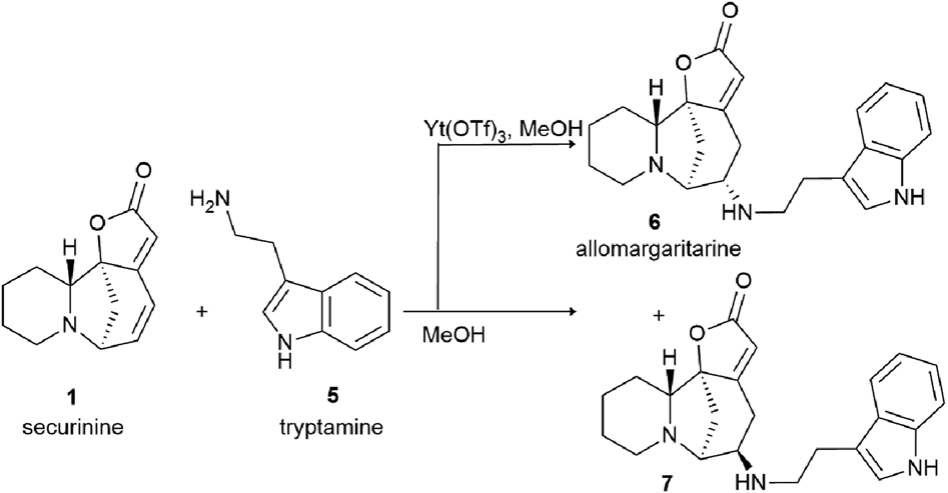
Stereospecific synthesis of allomargaritarine.

The system of conjugated double bonds of azobicyclooctane and lactone rings is of the greatest interest in terms of production of modified derivatives of securinine. Since this system is activated by a carbonyl group, it is easily attacked by nucleophiles. As a result of nucleophilic addition of amines to securinine, hybrid molecules containing a fragment of the alkaloid securinine and various pharmacophoric amines can be obtained. Thus, a stereospecific method for the production of amino derivatives of securinine^14,30,31,33–35^ was developed under the conditions of Lewis acid catalysis (Scheme 1). Various primary, secondary, and cyclic amines have been used to obtain amino derivatives.

**Fig. 4:**
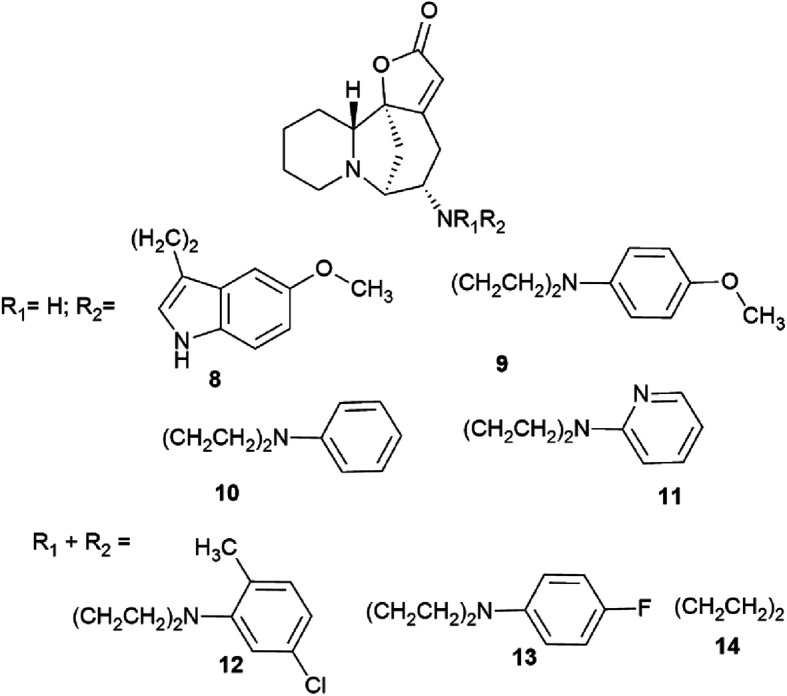
Scheme of the production of stereospecific amino derivatives of securinine under the conditions of Lewis acid catalysis.

A number of products of amination of securinine with various pharmacophoric amines were obtained under the conditions of Lewis acid catalysis (Fig. 4, 10–14) with obtaining only one stereoisomer.

Among the synthesized amino derivatives, the most challenging are tryptamine derivatives of securinine, in particular allomargaritarine 6, an isomer of the minor alkaloid margaritarine isolated from the plant *Margaritaria indica* Dalz.^36^ The replacement of the multiple bond with sp^3^-hybridized carbon atoms and the presence of an additional asymmetric center lead to a certain conformational mobility of the allomargaritarine molecule, which increases its possible interaction with the active receptor centers. As the studies have shown, allomargaritarine can be considered as a leader compound among the synthesized derivatives. Thus, allomargaritarine effectively suppresses Fe^3+^-induced LPO, probably due to the presence of iron-chelating activity.^34,37^ It has no effect on the mitochondrial transmembrane potential in the presence of the respiratory chain substrates and dose-dependently inhibits Ca^2+^-induced “swelling” of rat brain mitochondria, that is, allomargaritarine exhibits pronounced mitoprotective properties.^30^ Allomargaritarine, which has an antioxidant effect, effectively and concentration-dependently inhibits the aggregation of β-amyloid 1–42 during 24 hour incubation. Securinine not having antioxidant properties does not exert any inhibitory activity against β-amyloid aggregation.^31^ It has been shown that incubation of rat cerebral cortex cells with 25 μM allomargaritarine in various types of cellular neurotoxicity (glutamate toxicity, iron-induced toxicity, and Aβ-induced toxicity) significantly increases the percentage of surviving cells, in contrast to securinine. This may be due to both the antioxidant effect of allomargaritarine and its ability to increase mitochondrial resistance to the induction of mitochondrial permeability transition.^35^

It is known that securinine is an antagonist of GABA receptors^13,14^ and has been previously used as a neurostimulating agent in the treatment of various diseases, in particular, amyotrophic lateral sclerosis. However, its undesirable side effect, the pro-convulsive action, is also associated with the effect on the same target. In *in vivo* experiments, administration of securinine to mice at a dose of 20 mg kg^−1^ led to severe convulsions. With the introduction of allomargaritarine at a dose of 20 mg kg^−1^, no seizures were observed, and the mouse condition was characterized as stage 0 or 1, that is, allomargaritarine does not have pro-convulsive activity, unlike securinine. The studies of allomargaritarine effects in mice on the development of seizures in the pentylenetetrazol model of epilepsy showed that the long-term administration of allomargaritarine (5 mg kg^−1^ for 10 days) led to the decrease in the latent period of pentylenetetrazol-induced seizures of the third stage but did not affect their total duration. In the lithium–pilocarpine model of status epilepticus, allomargaritarine also increases the latency of seizures, but does not affect their duration.^38^ Thus, allomargaritarine can be considered as a promising neuroprotective drug with a complex nature of action.^31^

**Scheme 2:**
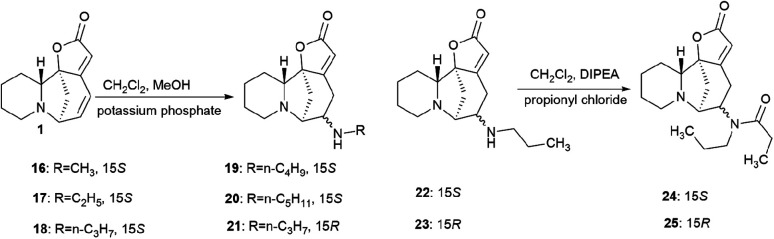
Scheme of the production of Michael adducts at the C14–C15 double bond.

Securinine conjugates with amines by the Michael addition reaction with the corresponding primary amines in the presence of potassium phosphate; as a result, conjugates 16–21 (Scheme 2) were obtained in good yields (68–78% yield).^39^

The disadvantage of the proposed method for the production of amino derivatives of securinine is the lack of stereospecificity: both *S* and *R* isomers are obtained. The authors note that the obtained amino derivatives have neuritogenic activity, but their activity is significantly lower than that of bivalent securinine analogues.^39^

**Scheme 3:**
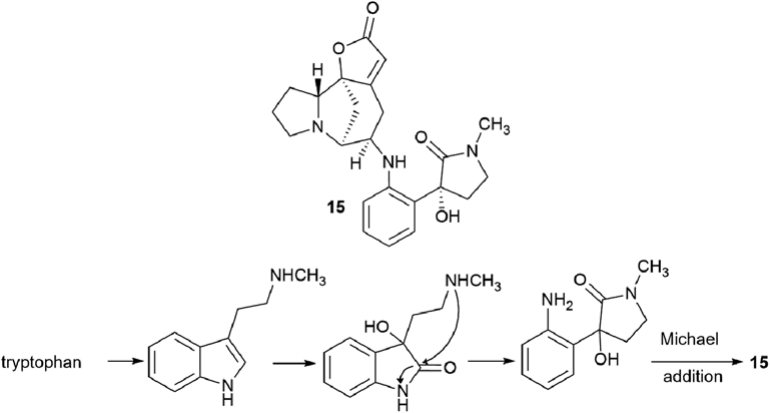
Structure of norsecurinamine A and the putative pathway of its biosynthesis.

Interestingly, another alkaloid was isolated from the fruits of the *Flueggea virosa* (Roxb. ex Willd.) Royle plants contain a fragment of norsecurinine and the pharmacophore amine donaxaridine, norsecurinamine A (15)^40^ (Scheme 3). The authors assumed a biosynthetic pathway for the formation of norsecurinamine A from norsecurinine and a key intermediate A *via* the Michael addition reaction. Intermediate A could be yielded from *N*-methyltryptamine, a co-existent tryptophan derivative isolated from the same plant material. Unfortunately, the authors only declared the isolation and establishment of the structure of norsecurinamine A but did not provide data on its biological activity.

### Stereoselective addition of 1,6-arylboronic acid conjugate to securinine

**Scheme 4:**
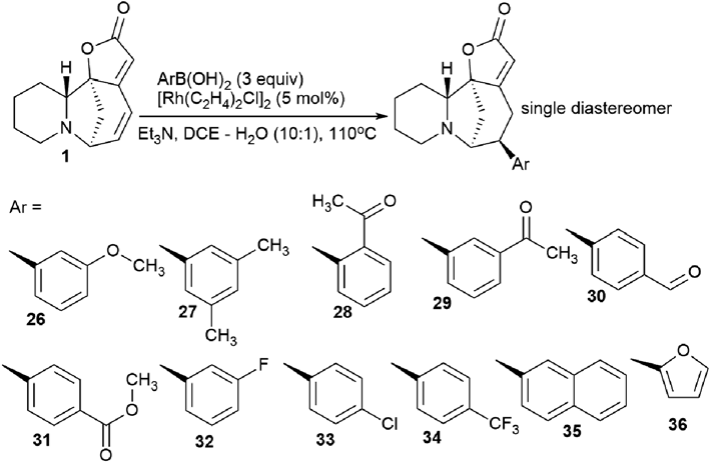
Stereoselective addition of 1,6-arylboronic acid conjugate to securinine.

The synthesis of securinine analogs carrying an aryl group at the C-15 position was performed.^41^ A rhodium-catalyzed 1,6-conjugate of commercially available arylboronic acids was added to securinine. Despite the complexity of this reaction,^42^ the authors managed to select the conditions and achieve high regio- and stereoselectivity of the resulting products. As a result, a number of securinine conjugates with an aryl fragment were obtained (Scheme 4). However, their biological activity was not investigated.

### Suzuki or Sonogashira cross coupling reactions

Perez *et al.* described the synthesis of a series of new securinine derivatives at the C14–C15 position using the Suzuki or Sonogashira cross-coupling reactions.^43^

**Scheme 5:**
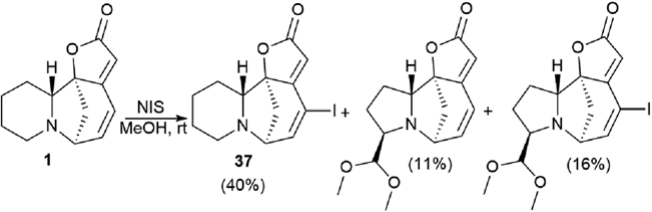
Synthesis of securinine derivatives. Production of iodine securinine derivative (37).

For the cross-coupling reaction, an iodine derivative of securinine was first obtained (Scheme 5, 37) according to the reported procedure.^44^

**Scheme 6:**
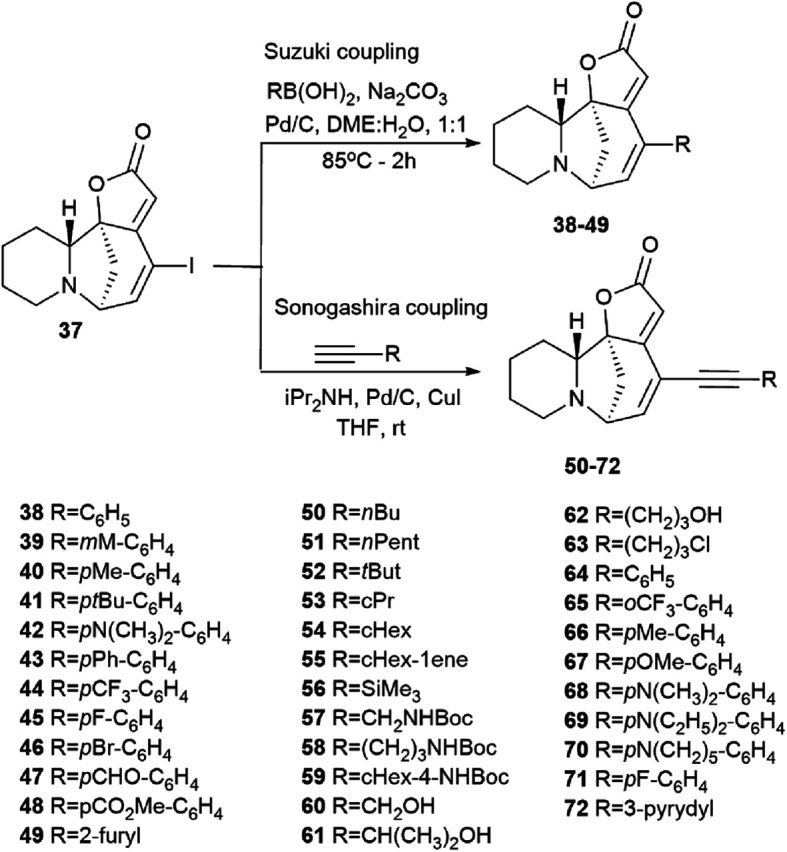
Synthesis of securinine derivatives by the Suzuki or Sonogashira cross-coupling reactions.

The first series was made by Suzuki cross-coupling with 37 and commercial boronic acids to get the target compounds 38–49 (Scheme 6). Using 1 mol% palladium on activated carbon as a catalyst, sodium carbonate as a base, in a mixture of water and 1,2-DME in a 1 : 1 ratio, various derivatives were obtained in high yields (from 25% to 93%) based on boric acids and securinine.

The results of determination of the cytotoxicity of 14-iodosecurinine 37 against the human colon cancer cell line HCT-116 showed the increase in cytotoxic activity of up to 80% at 1 μM, which suggests that diversification at the C14 position may lead to more active securinine derivatives. Unfortunately, most of these derivatives were found to be of little cytotoxicity. Indeed, only 43 and 44 were found to be weakly active, with tumor cell growth inhibition comparable to that of securinine. These results indicated that a spacer is needed between the securinine scaffold and the attached fragment to obtain more active derivatives. Based on the assumption, the authors performed the synthesis of compounds bearing an alkyne group as a spacer.

The second series of compounds were obtained by Sonogashira coupling^45–47^ between 37 and a set of commercially available acetylene derivatives to get the corresponding alkyne 50–72 (Scheme 6).^43^

**Fig. 5:**
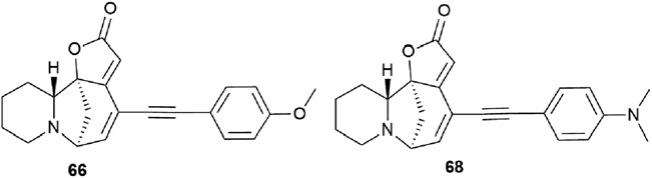
The most active compounds obtained by the Sonogashira cross-coupling reaction.

The cytotoxic properties of the obtained derivatives against the human colon cancer cell line HCT-116 were evaluated *in vitro* at concentrations of 20, 10 and 1 μM. Growth inhibition was measured after 72 hours of exposure to the compound. It turned out that the introduction of a phenyl group caused increased activity as evidenced by growth inhibition exceeding 50% even at 1 μM. Derivatives containing aliphatic substituents were also more cytotoxic than securinine, but to a lesser extent. The cyclohexyl derivative was found to be the strongest from the aliphatic series, comparable to the substituted phenyl series. These results indicate that lipophilic bulky substituents appear to be beneficial for enhanced cytotoxic properties. The carbamate derivative 45 has demonstrated as high cytotoxic activity as that of the 64–72 compounds, which represents an additional and attractive opportunity for the production of a new series of active compounds. The most cytotoxic compounds were tested in four tumor cell lines A-375 (melanoma), A549 (lung), HCT-116 (colon), and HL-60 (leukemia). Among all tested compounds, derivatives 66 and 68 (Fig. 5) exhibited the strongest cytotoxic activity to all four cell lines with the increase of at least 1 order of magnitude compared to the initial securinine, reaching nanomolar concentrations.^43^

A significant disadvantage of the derivatives obtained by Sonogashira cross-coupling is the lack of stability; for *in vivo* experiments, such derivatives should undergo further chemical optimization. Such modifications have recently been reported in the studies of antifolate compounds with a chemically stable alkyne bond.^48^

Compounds resulting from the Suzuki cross-coupling reaction with an aryl moiety at position C14 may be considered inactive on the HCT-116 colon cancer cell line. However, the introduction of an acetylene linker at the C14 position by Sonogashira cross-coupling^49^ led to an increase in activity against the same cancer cell line. It resulted in patent compounds 66 and 68 (Fig. 5) as antitumor agents.^50^

Securinine conjugates with amines by the Michael addition reaction were also produced.^39^ The Michael reaction with the corresponding primary amines was carried out in the presence of potassium phosphate; as a result, conjugates 16–21 were obtained in good yields (68–78% yield). Compounds 22–25 were synthesized by the acylation reaction of diacyl chlorides with the corresponding compounds (Scheme 2).

### Heck reaction

Challenging securinine derivatives were obtained by the Heck reaction using commercially available iodoarenes.^41^

**Scheme 7:**
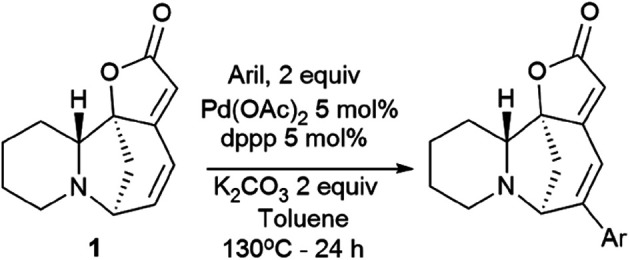
Synthesis of securinine derivatives obtained by the Heck reaction.

**Fig. 6:**
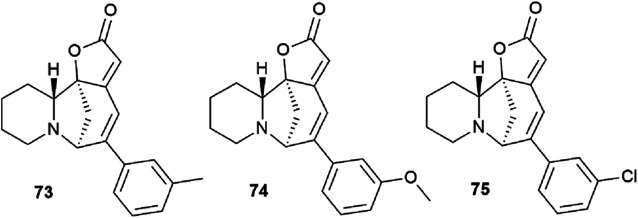
The most active securinine derivatives synthesized by the Heck reaction.

Securinine (1) reacted with functionalized aryl iodides in the presence of 5 mol% palladium acetate bound to dppp (1,3-bis(diphenylphosphino)propane) as a ligand and potassium carbonate as a base in toluene at 130 °C for 24 hours (Scheme 7). Under these conditions, the reaction proceeded with high regioselectivity in all cases, giving the target derivatives in good yields. Indeed, the reaction carried out with electron-rich aryl iodides led to the formation of the corresponding C15 arylated products in 36 to 97% yields. Similar results were obtained with electron-poor aryl iodides, with yields ranging from 52 to 85%. It should also be noted that the reaction does not proceed with *ortho*-substituted iodobenzene derivatives, probably due to the increased steric hindrances between the catalyst and the *ortho*-substituted group of the substrate during the reaction. The *in vitro* cytotoxicity of the new securinine analogues was first evaluated against the HCT-116 colon cancer cell line at concentrations of 20, 10 and 1 μM. The aromatic ring substitution was found to have a strong effect on cytotoxic activity. Indeed, while compounds with a substituent in the *para* position were weakly active, compounds bearing a substituent in the *meta* position (Fig. 6) showed better cytotoxicity than that of the parent securinine.

The most effective analogues of arylated securinine at the C15 position were tested on the HCT-116 cell line, as well as on three additional cancer cell lines, namely, A375 (melanoma), A549 (lung) and HL-60 (leukemia) with IC_50_ determinations. These compounds show an IC_50_ of 70 nM (Fig. 2), which corresponds to an 80-fold increase in potency over parent securinine. The metabolic profile of the most active derivative (75) (Fig. 6) has shown acceptable plasma stability, which makes it a valuable scaffold for the development of new anticancer drugs.

## C12–C13 double bond reactions

**Scheme 8:**
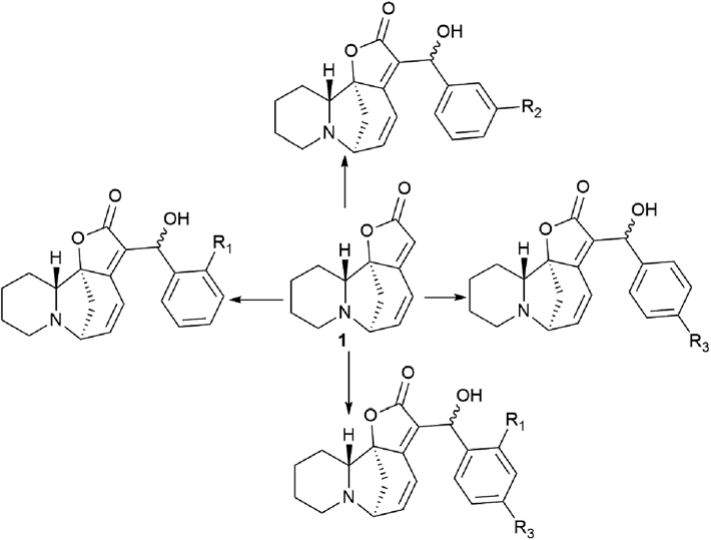
Securinine derivatives synthesized by the Baylis–Hillman reaction.

New securinine derivatives shown in Scheme 8 (ref. 51) were obtained using a non-stereoselective one-step Baylis–Hillman reaction^52–54^ between securinine and aromatic aldehydes, which are more reactive and susceptible to further derivatization than the corresponding alkylaldehydes.

**Scheme 9:**
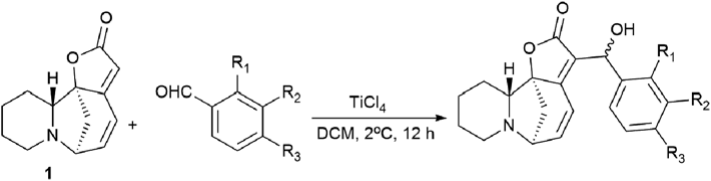
Derivatives containing a β′-hydroxy-α,β-unsaturated ketone moiety and with various substituents in the phenyl ring.

These derivatives were synthesized from securinine and various methylbenzaldehydes. In total, twenty-three derivatives containing a β′-hydroxy-α,β-unsaturated ketone moiety and various substituents including electron-withdrawing and electron-donating components in the phenyl ring were synthesized (Scheme 9).

**Scheme 10:**
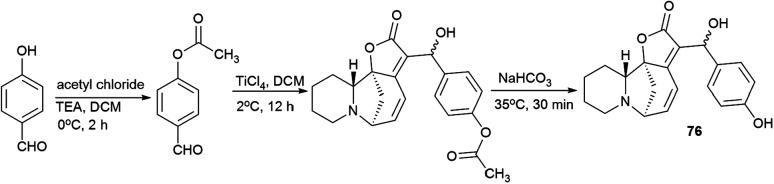
Synthesis of the 4-(hydroxymethyl)phenol derivative of securinine.

Since *p*-hydroxybenzaldehyde may form a complex with TiCl_4_, a three-step route shown in Scheme 10 was followed to obtain the compound (76). *p*-Hydroxybenzaldehyde reacted with acetyl chloride to obtain 4-(acetyloxy)-benzaldehyde, which was then converted by the Baylis–Hillman reaction into 4-(acetyloxy)-benzaldehyde securinine in the presence of TiCl_4_, from which the target product was obtained by deacetylation (76) (Scheme 10).

To investigate the possible effect of the configuration on the activity of a chiral carbon carrying a hydroxyl group, the resulting diastereomeric mixtures were separated by HPLC. The configurations of a chiral carbon carrying a hydroxyl group in diastereomers were identified by X-ray diffraction analysis. It turned out that all securinine derivatives were more effective inhibitors of Topo I than securinine itself. Based on the Topo I inhibition data, the relationship between the structure and activity of securinine derivatives can be summarized as follows: (1) the benzyl alcohol group is essential for the Topo I inhibitory activity; (2) substitution at the *para* position of the phenyl ring is better than the substitution at the *ortho* or *meta* position, and the compounds substituted at the *meta* position exhibit the least activity; (3) compared to single substitution in the *para* position, double substitution in both the *ortho* and *para* positions results in a slight decrease in activity; and (4) electron-withdrawing groups at the *para*-position of the phenyl ring are preferred over electron-donating groups. Diastereomers showed no obvious difference in their activity.^51^

The cytotoxic activity of all synthesized compounds against four human cancer cell lines, namely, A549, HeLa, HepG2, and SH-SY5Y, was also evaluated by an MTT test. Most securinine derivatives show higher cytotoxicity against HeLa, HepG2 and SH-SY5Y cell lines than that of camptothecin. The A549 tumor cell line is most sensitive to the action of the derivatives. The most active compounds were further tested against the L02 cell line (healthy hepatocyte). The results indicated that the compounds exhibit lower toxicity against human L02 hepatocytes than against cancer cell lines. Thus, it has been conclusively shown that the introduction of the β′-hydroxy-α,β-unsaturated ketone fragment into securinine improves the inhibitory activity against Topo I, as well as the antitumor activity of the derivatives.^51^

## Bivalent securinine analogues

There are many strategies for the synthesis of biologically active compounds. One of the current promising strategies being actively developed is the bivalent strategy. Accordingly, the synthesis is used for the production of compounds containing two identical pharmacophores connected with an estimated length and structure linker. This approach is often used in medicinal chemistry and drug development^55–58^ and has been successfully applied, for example, for the synthesis of bivalent bromodomain and extra-terminal motif-containing protein (BET) inhibitors,^59–61^ protein kinase inhibitors.^62^ The bivalent strategy was successfully used for the synthesis of antitumor agents based on natural compounds, for example, for the synthesis of dimers of artemisinin^63^ or podophyllotoxin,^64^ as well as other antitumor agents.^63,65–67^ The bivalent strategy has been successfully used for the synthesis of securinine derivatives. The starting point for the series of works was the isolation from the fruits of the plant *Flueggea virosa* (Roxb. ex Willd.) Royle, widely used in Chinese traditional medicine (Chinese name “bai fan shu”) for the treatment of rheumatism, itching, eczema, leukorrhea and injuries, contains dimeric analogues of securinine and norsecurinine.^68,69^ Later, another dimer of norsecurinine was isolated, norsecurinamine B,^39^ a symmetric dimer consisting of two molecules of norsecurinine alkaloids linked through the amino function and which was the first NH-bridged alkaloid oligomer derived from norsecurinine.

**Scheme 11:**
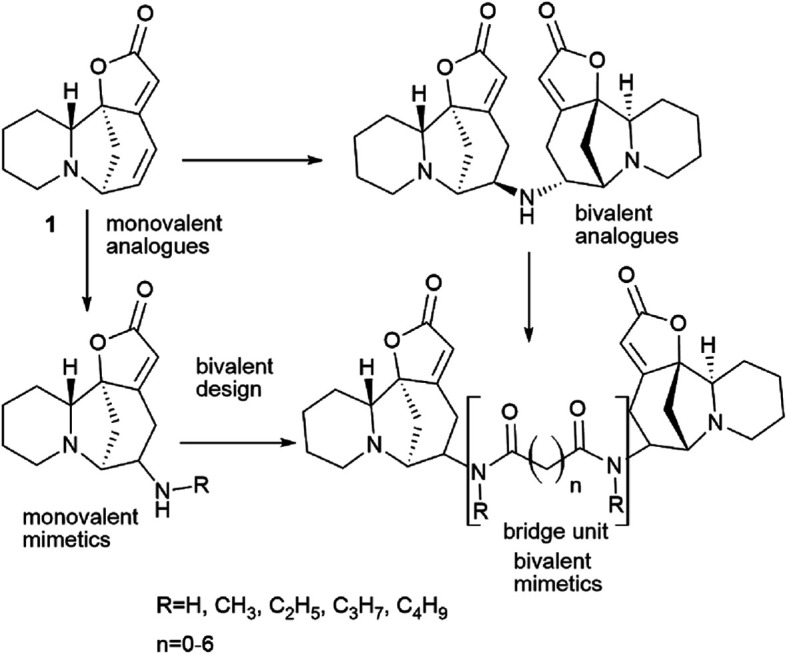
Design of bivalent securinine analogues.

**Scheme 12:**
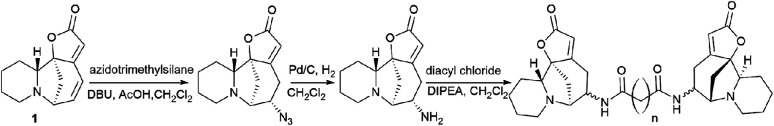
Synthesis of bivalent compounds based on securinine.

Thus,^39^*via* the bivalent approach often used in medicinal chemistry and drug development,^55–58^ a number of new monovalent and bivalent analogues of the securinine type were synthesized (Schemes 11 and 12).

The target bivalent compounds were synthesized, and various diacid amide chains were embedded in securinine dimeric analogues as bridging units. It suggests that bivalent mimetics may exhibit a stronger biological activity than that of monovalent mimetics.

The effect of synthesized compounds on neurite outgrowth was investigated. The study was performed on Neuro-2a neuroblastoma cells. The differentiation rate and length of common neurites of each cell were measured. It has been found that the preferred linker length between the two monomers is 4–7 carbon atoms, and the optimal side chain on the N atoms is ethyl or propyl. The SN3-L6 compound (77), one of the most active compounds, was selected as the leader compound, and its molecular action was investigated.^39^

The differentiation of neurons requires activation of several signaling pathways, including mitogen-activated protein kinase (MAPK), phosphoinositide 3-kinase (PI3K)–Akt, and Ca^2+^-dependent pathways.^70–74^ It turned out that when Neuro-2a cells were treated with the SN3-L6 compound (77), kinases 1/2 regulated by the extracellular signal (ERK1/2) were noticeably activated. Amino-terminal kinases c-Jun (JNK) are only temporarily activated after 5 min treatment, while the activity of p38 is not affected by SN3-L6 (77). The SN3-L6 compound (77) also significantly induces the activation of Akt and Ca^2+^/calmodulin-dependent protein kinase II (CaMKII). Western blot analysis showed that ERK, Akt, and CaMKII were activated by SN3-L6 (77). In addition, Rac1 and JNK may also be involved in the effect of compound SN3-L6 (77) on neurite elongation. Taken together, these results indicate that the SN3-L6 compound (77) induces neuronal differentiation and neurite network expansion *via* activation of the MEK–ERK, PI3K–Akt, and CaMKII pathways.^39^

Additional research has shown that the SN3-L6 leader compound (77) induces neuronal differentiation through a translation-dependent mechanism.^75^ A study carried out on Neuro-2a neuroblastoma cells showed that the SN3-L6 compound (77) activated a group of neurogenic transcriptional regulators, as well as proteins involved in RNA processing, translation, and metabolism. mRNAs of proneural transcription factors Foxp1, Foxp4, Hsf1, and ETS domain – containing transcription factor (Erf) are molecular targets that are translationally activated by SN3-L6 (77) and control neuronal differentiation. Foxp1 and Foxp4 are expressed in the fore and spinal cord and regulate the differentiation of glutamatergic projection neurons and motor neurons.^76,77^ Ectopic expression of Foxp1 in neural stem/progenitor cells (NSPCs) has been used to induce motor neuron or dopaminergic neuron identities.^78^ At the same time, Hsf1 is important for the neurogenesis of hippocampal and olfactory neurons in adults, and Erf is a sensitive gene in RA-induced neuronal differentiation.^79^ The authors conclude that the compound SN3-L6 (77) can act through the ERK–mTORCI–eEF2 pathway and can be used to activate translation and neuronal differentiation.^75^

**Scheme 13:**
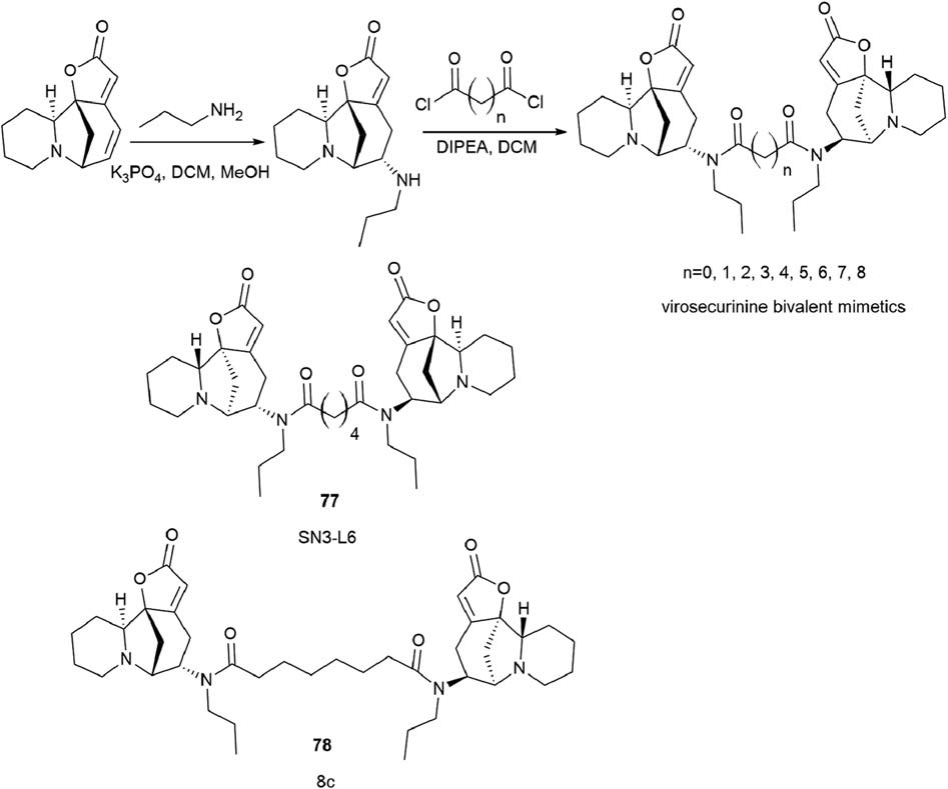
Synthesis of bivalent analogues of virosecurinine and the most effective compounds SN3-L6 (77) and 8c (78).

To continue this work, a series of bivalent derivatives of virosecurinine were synthesized in order to investigate the possible differences in biological activity arising from different stereochemistries.^80^ The previously obtained series of securinine bivalent mimetics with a diamide linker^39^ and series of new virosecurinine bivalent mimetics (Scheme 13) were investigated as effective reversal agents against P-glycoprotein-mediated multidrug resistance.^80^

As it is known, chemotherapy is the main therapy for cancer, and its most serious obstacle is multidrug resistance (MDR).^81–84^ ABCB1 (P-gp)-mediated drug resistance is the most common, and P-glycoprotein is seen as a promising molecular target for overcoming MDR.^85–87^ In cancer treatment, it may contribute to MDR development as it pumps chemotherapeutic agents out of cells.^88^ Many drugs are P-gp substrates including anthracycline antibiotics, and taxol derivatives.^84,89–91^ Accordingly, bivalent securinine mimetics have been investigated as P-gp modulators. It turned out that they exhibit high activity as modulators of P-gp. Leader compound 8c (78) has obvious MDR reversal activity *in vitro* and *in vivo*.^80^ The administration of 10 μM compound 8c (78) almost completely removes doxorubicin (DOX) resistance in both HepG2/DOX and MCF-7/ADM cell lines more effectively than verapamil. This is also confirmed by *in vitro* experiments: subcutaneous administration of 25 mg kg^−1^ 8c (78) showed high activity in reversal of doxorubicin resistance in the HepG2/DOX xenograft model.^80^

**Fig. 7:**
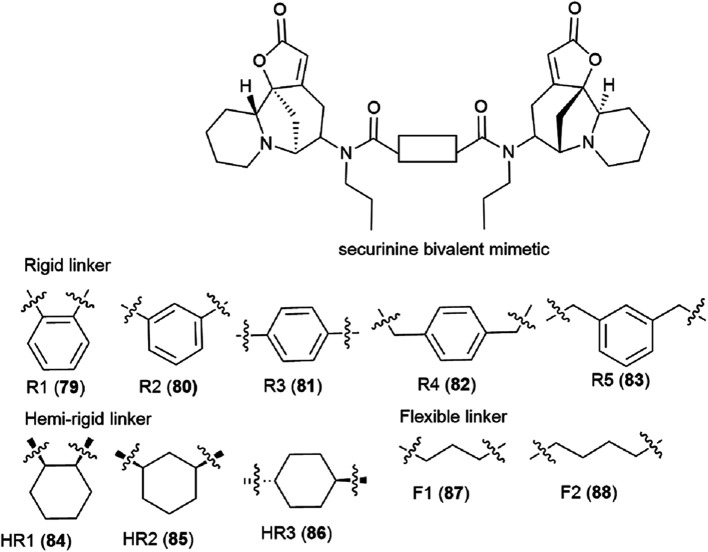
Design of bivalent securinine mimetics with various linkers.

It is clear that the linker structure between two securinine molecules influences the activity of bivalent mimetics. The work^92^ describes the synthesis of bivalent mimetics with rigid, semi-rigid, and flexible linkers between the C-15 and C-15′ atoms. The design strategy for bivalent mimetics with different linkers is shown in Fig. 7.

**Scheme 14:**
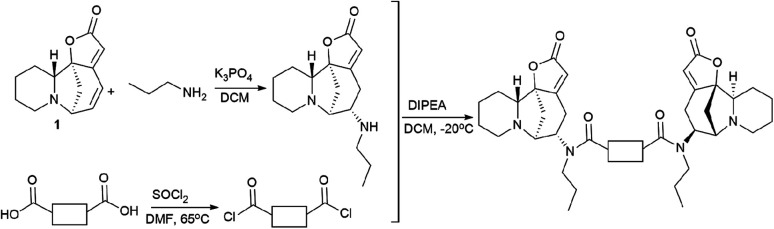
Scheme for the synthesis of bivalent securinine mimetics with various linkers.

For that, a propylamine securinine derivative was obtained with the hetero-Michael addition reaction^93^ (Scheme 14). Then, the corresponding diacid-containing specified linker was added to thionyl chloride, and thus the diacid chlorides reacting with the propylamine derivative of securinine were produced.

Thus, 12 bivalent securinine mimetics were obtained. The effect of bivalent securinine mimetics on topoisomerase 1 activity was investigated in several tests, docking was performed, and mechanistically relevant assays were performed. The studies have shown a significant increase in the inhibitory activity of bivalent mimetics compared to the original securinine and monovalent derivatives. All bivalent rigid linker mimetics were more potent inhibitors than securinine itself, with the most effective compound R2 (80) being comparable in potency to the control camptothecin. Bivalent semi-rigid linker securinine mimetics are less effective, while flexible linker compounds have shown no inhibitory activity. In particular, the conformationally limiting (rigid) linker is decisive as evidenced by the decrease in activity when shifted from the phenyl-based group to the cyclohexyl-based group, and then to the propyl linker. The relative position of securinine units is also important: the activity increases from *ortho*- to *meta*-disubstituted linkers.

These results indicate that the presence of a second unit of securinine may confer increased activity, but the way how the two units are linked is crucial. As shown by the docking of the most active compound R2 (80) with Topoisomerase I, the binding of the bivalent mimetic in the active pocket of the enzyme to residues ARG362, ARG364, LYS374, and ASN722 precludes the normal interaction between Topoisomerase I and DNA. A similar effect is observed upon docking of compound R2 (80) with the Topo I/DNA covalent complex through six hydrogen bonds with two neighboring DNA bases (DA113 and DA114) and the LYS425 residue of Topo I. As a result of these studies, an effective and promising inhibitor of Topoisomerase 1 was identified: a bivalent securinine mimetic with a rigid linker (compound R2 (80)).^92^

**Fig. 8:**
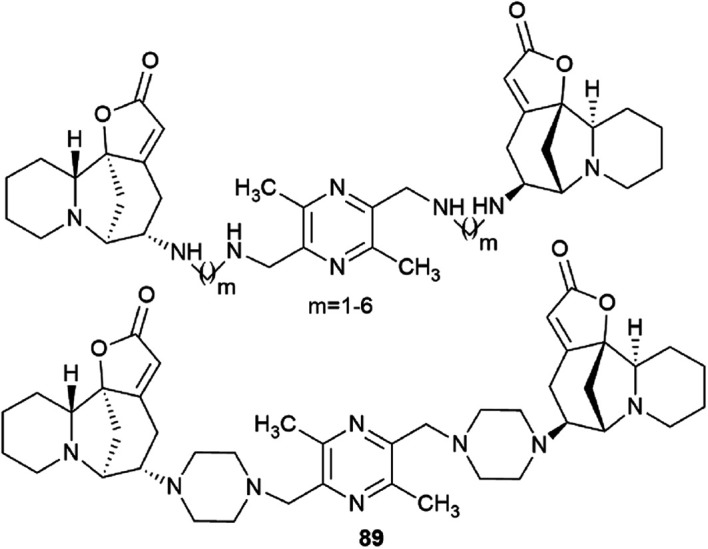
Design of bivalent securinine mimetics using ligustrazine as a linker.

Challenging securinine dimers with high antitumor activity are described in the patent^94^ (Fig. 8). Ligustrazine was used as the central linker linking two securinine molecules.

Ligustrazine is one of the main biologically active components known from the traditional Chinese medicine Chuanxiong (*Ligusticum wallichii* Franchat), widely used for the clinical treatment of cardiovascular diseases,^95–97^ and has pronounced antitumor^98^ and neuroprotective properties.^99–101^ Numerous series of derivatives have been synthesized on the basis of ligustrazine including dimeric ones.^100,102,103^

**Scheme 15:**
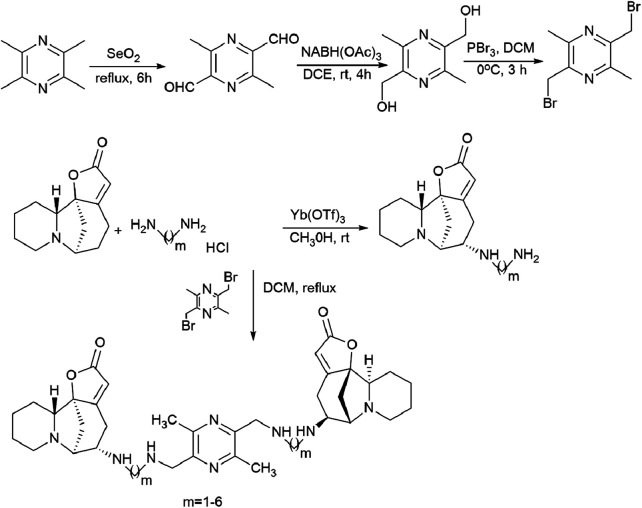
Synthesis of bivalent compounds based on ligustrazine and securinine.

The patent^94^ describes in detail the synthesis of bivalent compounds based on ligustrazine and securinine (Scheme 15) with the preliminary production of 2,5-bis(bromomethyl)-3,6-dimethylpyrazine and aminosecurinine followed by their combination into a bivalent derivative.

Thus, the use of a bivalent strategy for the synthesis of securinine derivatives is one of the promising trends for the production of active compounds.

## Conclusions

Securinine is a unique indolizidine alkaloid combining four cycles, 6-azobicyclo[3.2.1]octane as the key structure fused with α,β-unsaturated-γ-lactone and piperidine, and is a rigid molecule. It has several reactive centers: first of all, these are double bonds at positions 12–13 and 14–15. The presence of these centers opens wide opportunities for the chemical modification of securinine and the production of more conformationally flexible molecules. A number of studies have shown both neuroprotective and antitumor activities of securinine derivatives. Attention is drawn to the fact that securinine derivatives were studied as only antitumor or only neuroprotective compounds. However, it is clear that these compounds have a broader potential and should be more investigated.

Attention is drawn to the technology for the production of bivalent securinine conjugates. Using different length, structures of the linker, and the nature of the connection of securinine fragments, it is possible to increase both the specific activity and the production of derivatives with other activities. These are bivalent securinine conjugates that seem to be the most promising, in our opinion, and have the potential for further research.

Thus, securinine is a promising scaffold for the development of neuroprotective and anticancer drugs with a broad spectrum of action.

## Conflicts of interest

There are no conflicts to declare.

## Supplementary Material
